# Melanization in response to wounding is ancestral in arthropods and conserved in albino cave species

**DOI:** 10.1038/s41598-017-17471-2

**Published:** 2017-12-07

**Authors:** Helena Bilandžija, Mara Laslo, Megan L. Porter, Daniel W. Fong

**Affiliations:** 1 Department of Molecular Biology, Ruđer Bošković Institute, Zagreb, 10000 Croatia; 2 Museum of Comparative Zoology, Department of Organismic and Evolutionary Biology, Harvard University, Cambridge, MA 02138 USA; 3 Department of Biology, University of Hawai’i at Mānoa, Honolulu, HI 96822 USA; 4 Department of Biology, American University, Washington, DC 20016 USA

## Abstract

Many species adapted to aphotic subterranean habitats have lost all body pigmentation. Yet, melanization is an important component of wound healing in arthropods. We amputated appendages in a variety of cave-adapted and surface-dwelling arthropods. A dark clot formed at the site of injury in most species tested, including even albino cave-adapted species. The dark coloration of the clots was due to melanin deposition. The speed of wound melanization was uncorrelated with a difference in metabolic rate between surface and cave populations of an amphipod. The chelicerate *Limulus polyphemus*, all isopod crustaceans tested, and the cave shrimp *Troglocaris anophthalmus* did not melanize wounds. The loss of wound melanization in *T. anophthalmus* was an apomorphy associated with adaptation to subterranean habitats, but in isopods it appeared to be a symplesiomorphy unrelated to colonization of subterranean habitats. We conclude that wound melanization i) is an important part of innate immunity because it was present in all major arthropod lineages, ii) is retained in most albino cave species, and iii) has been lost several times during arthropod evolution, indicating melanization is not an indispensable component of wound healing in arthropods.

## Introduction

The loss of body and eye pigmentation, or albinism, is a convergent feature found in a majority of species adapted to aphotic subterranean habitats, such as caves^1^. However, the evolutionary causes of albinism in cave organisms have not been fully explored, with only a few studies investigating molecular mechanisms underlying loss of pigmentation^2–4^. A longstanding idea is that relaxed selection for pigmented traits in darkness allows for accumulation of mutations that eventually eliminate pigment production. Indeed, functions of pigments, such as protection from harmful UV radiation, camouflage or aposematic coloration for escaping predation, attracting potential mates, etc., are unnecessary in aphotic environments. However, pigments or pigment synthesis pathways can have pleiotropic roles in different metabolic and physiological processes, such as scavenging of reactive oxygen species, physical protection, temperature regulation and innate immunity^5–8^. For example, blocking melanin synthesis may enable precursor molecules to be used in other metabolic pathways. In the fish *Astyanax mexicanus*, silencing of the *oca2* gene in the surface form caused a loss of pigmentation and an increase of both the melanin precursor L-tyrosine as well as the catecholaminergic neurotransmitter dopamine^9^. Furthermore, the levels of catecholamines (CAT) are higher in *Astyanax* cavefish compared to the surface form^9^. CATs are involved in numerous physiological functions and behaviors. A suite of traits related to the CAT system were changed as cavefish evolved from surface-dwelling ancestors, including feeding behavior, stress response, and sleeping patterns, among others^10–12^. Therefore, the loss of melanin pigmentation might be adaptive in light depleted environments, but does it come with a cost?

The innate immune system is the only means of immunity in invertebrates, although there is some controversy surrounding the recent discovery of innate priming or innate memory as a type of adaptive immunity in invertebrates^13^. In arthropods, innate immunity consists of cellular and humoral defenses such as phagocytosis, nodulation and encapsulation, synthesis of antimicrobial peptides, production of reactive intermediates of oxygen and nitrogen, release of stress associated proteins and substances that function in opsonization and iron sequestration, and complex proteolytic cascades that culminate with coagulation, clotting and melanin formation^7,14^. Melanization has multiple roles in the immune response, including encapsulation of pathogens and parasites, wound healing, clot formation and production of cytotoxic intermediates that kill invading microorganisms^15,16^. Melanin also adds to the stiffness of clots, preventing the loss of hemolymph and invasion of pathogens into the hemocoel^17^.

Part of the melanin synthesis pathway is catalyzed by the enzyme phenoloxidase (PO). Normally phenoloxidase exists as the proenzyme prophenoloxidase (PPO) that is proteolytically activated by a cascade of serine proteases. Activation of PO must be tightly controlled temporally and spatially because intermediate products of its action are severely cytotoxic. PO catalyzes hydroxylation of phenolic substrates into o-diphenols and their subsequent oxidation into quinones, which polymerize into melanin. PPO is stored in cytoplasmic granules of hemocytes and is released into the hemolymph following the activation of pattern recognition proteins upon detection of bacterial or fungal antigens or both^14,17,18^. PO is extremely important in arthropods. PO functions in both exoskeleton formation as well as in effective immune system responses, and simultaneous appearance of these two processes is thought to have fostered the remarkable radiation of arthropods^19^.

In this study, we attempted to determine if cave arthropods lacking body pigmentation have also lost the melanization part of the innate immune response. This is not a trivial question because although the ability to immediately respond to an immune challenge is critical for survival, maintenance of parts of the innate immunity is costly in terms of resources^7,20^. Tyrosine is a critical component of the PO mediated melanin synthesis pathway, and it is derived from phenylalanine, which is obtainable only from ingested food^21^, while melanin is itself a nitrogen rich compound and requires significant input of protein rich resources for its synthesis^22^. Subterranean environments are relatively resource poor compared to surface environments primarily because of the lack of photosynthesis, and are generally nitrogen rather than carbon limited^1^; thus, it is conceivable that adaptations to such resource limited environments may require a compromise in resource intensive physiological systems. Subterranean environments are hypothesized to harbor lower abundance and diversity of parasites and pathogens than surface environments (e.g.^23^), and cave organisms may experience relaxed selection to maintain the highly resource demanding portion of the innate immune system such as the melanin synthesis pathway. We found that most albino cave arthropods retain the ability to melanize wounds, and that PO is involved in this response. We also found that the wound melanization response is highly conserved across arthropod taxa, but has been lost in isopods, the chelicerate *Limulus polyphemus* and the cave shrimp *Troglocaris anophthalmus*.

## Materials and Methods

### Animals used

Arthropods of diverse taxa belonging to multiple higher taxonomic groups were collected periodically between 2013 and 2017 from cave and surface habitats in Bosnia and Herzegovina, Croatia, and the United States, with special focus on albino subterranean species and their surface-dwelling relatives, including multiple geographically separated populations or genetically distinct clades of some of the species (see Tables 1 and 2). Most of the species belonged to the crustacean orders Amphipoda, Decapoda and Isopoda, but a variety of other higher taxonomic groups were included. After collection, the live animals were brought to laboratories or field facilities and maintained in conditions that closely resembled those of their natural habitats, including similar temperatures, high humidity for terrestrial organisms and native water for aquatic species. Experiments were performed after the animals had acclimated to the captive conditions from several hours up to three days.

**Table 1:** Higher taxonomic groupings and results of wound melanization assay for each species.

Class	Order	Family	Species (albinos in bold*)	WM	PTU
Pycnogonida	Pantopoda	Phoxichilidiidae	*Anoplodactylus lentus*	1/1	
Merostomata	Xiphosura	Limulidae	*Limulus polyphemus*	**0/1**	na
Arachnida	Araneae	Dysderidae	*Stalagtia hercegovinensis*	1/1	
	Oribatida	Trhypochthoniidae	*Archegozetes longisetosus*	3/3	
	Pseudoscorpionida	Neobisiidae	*Neobisium sp*.	5/5	
	Scorpiones	Euscorpiidae	*Euscorpius sp*.	1/1	
Diplopoda	Chordeumatida	Cleidogonidae	Pseudotremia fulgida*	3/3	2/2
			*P. hoffmani*	1/1	
	Polydesmida	Polydesmidae	Brachydesmus inferus*	4/4	
			Brachydesmus sp.*	5/5	3/3
Malacostraca	Amphipoda	Caprellidae	*Caprella sp*.	4/4	
		Crangonyctidae	Bactrurus brachycaudus*	3/3	1/1
			Crangonyx antennatus*	4/4	2/2
			*C. forbesi a*	6/6	3/3
			*C. forbesi b*	6/6	6/6
			*C. shoemakeri*	6/6	3/3
			Stygobromus conradi*	3/3	2/2
			S. emarginatus* a	6/6	3/3
			S. emarginatus* b	2/2	1/1
			S. flagellatus*	3/3	3/3
			S. leensis*	2/2	1/1
			S. tenuis potomacus*	6/6	3/3
		Gammaridae	Gammarus cohabitus*	2/2	
			*G. minus a*	6/6	6/6
			*G. minus b*	6/6	6/6
			G. minus* c	3/3	3/3
			G. minus* d	6/6	6/6
		Niphargidae	Niphargus sp.* a	6/6	
			Niphargus sp.* b	1/1	
			Niphargus sp.* c	3/3	
	Decapoda	Atyidae	*Atyaephyra desmarestii*	5/5	
			Troglocaris anophthalmus* a	**0/1**	na
			T. anophthalmus* b	**0/5**	na
			T. anophthalmus* c	**0/3**	na
			T. anophthalmus* d	**0/4**	na
		Crangonidae	*Crangon sp*.	3/3	3/3
		Palaemonidae	Palaemon antrorum*	6/6	2/2
			*P. paludosus*	3/3	
	Isopoda	Asellidae	*Asellus aquaticus*	**0/4**	na
			*Caecidotea kenki*	**0/6**	na
			C. bicrenata bicrenata*	**0/3**	na
			C. pricei* a	**0/6**	na
			C. pricei* b	**0/3**	na
			*Lirceus brachyurus*	**0/6**	na
			Proasellus hercegovinensis*	**0/3**	na
			Proasellus sp.*	**0/3**	na
		Cirolanidae	Cirolanides texensis*	**0/2**	na
		Trichoniscidae	Alpioniscus balthazari*	**0/5**	na
			Titanethes albus*	**0/3**	na
		Sphaeromatidae	Monolistra caeca*	**0/5**	na
			Monolistra sp.*	**0/2**	na
			Sphaeromides virei* a	**0/1**	na
			S. virei* b	**0/1**	na
Collembola	Entomobryomorpha	Entomobryidae	*Entomobrya sp*.	4/4	1/1
		Isotomidae	*Folsomia candida*	1/1	
Insecta	Hemiptera	Cixiidae	Oliarus polyphemus*	5/5	2/2
	Coleoptera	Carabidae	*Neotrechus sp*.	2/2	

Habitat and collection location information are given in Table 2. Albino species are given in bold and marked with an asterisk. Each occurrence of a species listed more than once, differentiated with a letter after the species name, represents a distinct geographic population or clade (see Table 2). WM = wound melanization response, given as number of individuals showing melanized wounds/number of individuals with induced wounds; negative responses are given in bold. PTU = number of individuals where wound melanization was blocked by PTU treatment/number of individuals treated; na denotes not applicable to species showing no wound melanization; blank denotes PTU treatment was not attempted due to lack of specimens or PTU being unavailable in field facilities.

**Table 2:** Habitats and origins of species included in this study.

Species (albinos in bold*)	Habitat	Collection Site	County/Region	US State/Country
*Anoplodactylus lentus*	Littoral zone	Woods Hole	Barnstable	Massachusetts
*Limulus polyphemus*	Littoral zone	Woods Hole	Barnstable	Massachusetts
*Stalagtia hercegovinensis*	Cave wall	Tučepska vilenjača (cave)	Middle Dalmatia	Croatia
*Archegozetes longisetosus*	Moss/leaf litter	Lab-bred colony	Middlesex	Massachusetts
*Neobisium sp*.	Cave wall	Baba (cave)	Middle Dalmatia	Croatia
*Euscorpius sp*.	Cave floor	Jama pod Stipkovcem (cave)	Middle Dalmatia	Croatia
Pseudotremia fulgida*	Cave wall	Buckeye Creek Cave	Greenbrier	West Virginia
*P. hoffmani*	Cave wall	Buckeye Creek Cave	Greenbrier	West Virginia
Brachydesmus inferus*	Cave wall	Tamnica (cave)	Kordun	Croatia
Brachydesmus sp.*	Cave wall	Miljacka II (cave)	Northern Dalmatia	Croatia
*Caprella sp*.	Littoral zone	Woods Hole	Barnstable	Massachusetts
Bactrurus brachycaudus*	Interstitial	Spring near Elsah	Jersey	Illinois
Crangonyx antennatus*	Cave pool	Gallohan Cave	Lee	Virginia
*C. forbesi a*	Surface Stream	Pallisades	Jersey	Illinois
*C. forbesi b*	Surface Stream	Loutre River tributary	Montgomery	Missouri
*C. shoemakeri*	Seepage Spring	Great Falls	Montgomery	Maryland
Stygobromus conradi*	Epikarst	Marshall Cave	Highland	Virginia
S. emarginatus* a	Cave stream	Organ Cave	Greenbrier	West Virginia
S. emarginatus* b	Cave stream	Patton Cave	Monroe	West Virginia
S. flagellatus*	Phreatic aquifer	Artesian spring	Hays	Texas
S. leensis*	Epikarst	Litton Cave	Lee	Virginia
S. tenuis potomacus*	Hypotelminorheic	Seepage Spring	Arlington	Virginia
Gammarus cohabitus*	Karst spring	Tylersville Spring	Centre	Pennsylvania
*G. minus a*	Karst spring	Maiden Spring	Tazewell	Virginia
*G. minus b*	Karst spring	Ward Spring	Greenbrier	West Virginia
G. minus* c	Cave stream	Fallen Rock Cave	Tazewell	Virginia
G. minus* d	Cave stream	Organ Cave	Greenbrier	West Virginia
Niphargus sp.* a	Cave stream	Tamnica (cave)	Kordun	Croatia
Niphargus sp.* b	Cave stream	Mandelaja (cave)	Kordun	Croatia
Niphargus sp.* c	Cave pool	Bunar kod Rasline (cave)	Northern Dalmatia	Croatia
*Atyaephyra desmarestii*	Cave lake	Jama u Predolcu (cave)	Neretva	Croatia
Troglocaris anophthalmus* a	Phreatic aquifer	Bunar kod Rasline (cave)	Northern Dalmatia	Croatia
T. anophthalmus* b	Phreatic aquifer	Izvor špilja Karišnica (cave)	Northern Dalmatia	Croatia
T. anophthalmus* c	Cave lake	Jama u Predolcu (cave)	Neretva	Croatia
T. anophthalmus* d	Phreatic aquifer	Tamnica (cave)	Kordun	Croatia
*Crangon sp*.	Littoral zone	Woods Hole	Barnstable	Massachusetts
Palaemon antrorum*	Phreatic aquifer	Artesian spring	Hays	Texas
*P. paludosus*	Lakes and ponds	Commercial dealer	Howard	Maryland
*Asellus aquaticus*	Karst spring	Berberov buk	Northern Dalmatia	Croatia
*Caecidotea kenki*	Seepage Spring	Seepage Spring	Arlington	Virginia
C. bicrenata bicrenata*	Cave stream	Bucket O’Blood Cave	Sewanee	Tennessee
C. pricei* a	Karst spring	Caskey Spring	Berkeley	West Virginia
C. pricei* b	Cave stream	Odgen Cave	Frederick	Virginia
*Lirceus brachyurus*	Karst spring	White House Spring	Jefferson	West Virginia
Proasellus hercegovinensis*	Cave pool	Bjelušica (cave)	Southern Herzegovina	Bosnia and Herzegovina
Proasellus sp.*	Cave pool	Manita peć (cave)	Northern Dalmatia	Croatia
Cirolanides texensis*	Phreatic aquifer	Artesian spring	Hays	Texas
Alpioniscus balthazari*	Cave floor	Miljacka II (cave)	Northern Dalmatia	Croatia
Titanethes albus*	Cave floor	Mrgića špilja (cave)	Kordun	Croatia
Monolistra caeca*	Cave stream	Tamnica (cave)	Kordun	Croatia
Monolistra sp.*	Cave stream	Miljacka II (cave)	Northern Dalmatia	Croatia
Sphaeromides virei* a	Cave stream	Miljacka II (cave)	Northern Dalmatia	Croatia
S. virei* b	Cave lake	Izvor špilja Karišnica (cave)	Northern Dalmatia	Croatia
*Entomobrya sp*.	Leaf litter	Pimmit Run	Arlington	Virginia
*Folsomia candida*	Lab substrate	Lab-bred colony	Middlesex	Massachusetts
Oliarus polyphemus*	Lava tube	Kaumana cave	Hawaii	Hawaii
*Neotrechus sp*.	Cave floor	Jama pod Stipkovcem (cave)	Middle Dalmatia	Croatia

Species are listed in the same order as in Table 1 for cross reference. Albino species are given in bold and marked with an asterisk. Each occurrence of a species listed more than once, differentiated with a letter after the species name, represents a distinct geographic population or clade.

### Wound melanization assays

To determine whether the targeted arthropods could synthesize melanin after an immune challenge, we induced wounds by amputating one or more of the body appendages, usually walking legs or antennae or both. For small organisms with short appendages such as springtails and mites, we pierced the cuticle with a fine-pointed needle. Prior to the procedure, the animals were sedated by being placed in a freezer for two to three minutes for terrestrial species or with chemical anesthetics such as tricane or menthol crystals for aquatic species. After the operation animals were left to recover and periodically inspected for the appearance of a dark plug at the site of injury over the next several days. The dark cuticle of some species, such as Pycnogonida and the surface-dwelling isopod *Asellus aquaticus*, rendered it difficult to ascertain whether melanization of the wound had occurred even under the microscope. Amputated limbs of such species were treated for several hours with formic acid, which removed body pigments but does not dissolve melanin, and then inspected for presence of a dark plug at the wound site.

Some animals were treated with phenylthiourea (PTU), an inhibitor of phenoloxidase^24^, the enzyme catalyzing critical steps in the melanin synthesis pathway of invertebrates. The PTU treatment was terminated when black plugs appeared in the wound sites of specimens not treated with PTU, usually within 24 to 48 hours. If PTU prevented darkening of the wound sites, then the dark coloration of wound sites observed in specimens not treated with PTU was interpreted as resulting from melanin pigment synthesis and deposition. Aquatic species were treated with 0.1% PTU in native water, except albino crangonyctid amphipods (see Table 1) were treated with 0.05% PTU. Initial trials with concentrations higher than 0.05% and 0.1% resulted in mortality of some of the albino crangonyctid amphipods and in the other species, respectively. Terrestrial species were placed in small, covered containers with absorbent paper moistened with 0.1% PTU, except 0.25% PTU was used for diplopods (see Table 1), and sufficient amount of the paper was used to ensure contact between the tips of the amputated legs and the PTU infused paper when the animals moved. The higher PTU concentration was used for diplopods because they had numerous walking legs (>40) and thus we expected less frequent contact between the two to three amputated legs and the PTU infused paper compared to the one amputated leg among only six or eight legs possessed by the other terrestrial species treated; however, whether this resulted in delivery of similar dosage of PTU to the wound sites between these two sets of terrestrial species was unknown. Because the wound melanization response was inhibited in all specimens treated with PTU regardless of concentration and habitat, we deemed our usage of different concentrations did not bias our results.

A reduction in metabolic rate in cave-adapted taxa compared to related surface-dwelling taxa is considered an adaptation to the relatively food poor subterranean environment^1^. We therefore compared the speed of the wound melanization process among two surface and two cave populations of the amphipod *Gammarus minus* because: 1) the wound melanization assays on this species were performed in the laboratory rather than under field conditions, 2) this species exists as multiple morphologically distinct populations inhabiting the surface and cave habitats, and 3) different cave populations had independently evolved the cave phenotype^25^.

### Data availability statement

The datasets generated during and/or analysed during the current study are available from the corresponding author on reasonable request.

## Results

### Albino cave invertebrates retain the ability to synthesize melanin in response to wounding

**Figure 1:**
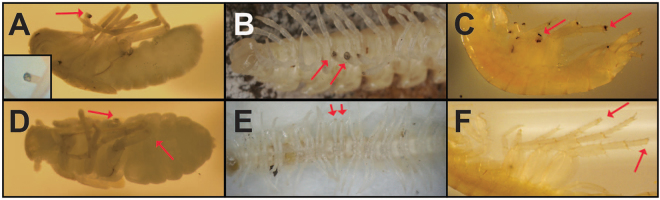
Albino cave invertebrates retain the ability to synthesize melanin in response to wounding. The site of cuticular injury turned dark in the cixiid *Oliarus polyphemus* (**A**), the diplopod *Brachydesmus inferus* (**B**), and the amphipod *Stygobromus emarginatus* (**C**). PTU treated specimens showed no black plug formation (**D**–**F**), suggesting phenoloxidase is involved in the melanization pathway. Inset in A is a magnified image of another *O. polyphemus* amputated leg. Red arrows point out the wound site. Photographs by the authors.

All albino subterranean invertebrate species we tested, except for isopods and the atyid shrimp *Troglocaris anophthalmus* (discussed further below), showed melanization of experimentally induced wound sites (Table 1. See Fig. 1 for results from a subset of the arthropods tested). These included the following: all 14 species and clades of amphipods (e.g., Fig. 1C) belonging to five genera in three families occupying a variety of subterranean habitats such as the hypotelminorheic, epikarst, cave streams and phreatic aquifers; the palaemonid decapod shrimp *Palaemon antrorum*; three millipede species in two orders, the cleidogonid *Pseudotremia fulgida* in the Chordeumatida and two polydesmid *Brachydesmus* species (Fig. 1B) in the Polydesmida; and an insect, the cixiid planthopper *Oliarus polyphemus* (Fig. 1A). Furthermore, the wound melanization process was inhibited in all 14 of the albino species in addition to all seven non-albino species that were treated with PTU (Table 1, see also Fig. 1D–F). These results demonstrated for the first time that a diverse group of albino cave-adapted arthropod species from a variety of subterranean habitats, all showing no cuticular body pigment, retained the ability to synthesize melanin during wound healing, and that phenoloxidase played an important role in this reaction in both albino and non-albino species.

### The speed of wound melanization is similar between cave-adapted compared to surface-dwelling populations of the amphipod *Gammarus minus*

We compared the speed of formation of the melanized plug at tips of amputated appendages among two surface-dwelling and two cave-adapted *Gammarus minus* populations as well as *G. cohabitus*, the *Gammarus* species with the most cave-adapted morphology^26^. The wound healing process proceeded as follows. About 6 hours post amputation (hpa), a whitish plug had formed but it was only loosely bound to the wound site because the entire plug could be dislodged by gently swirling the water surrounding the animal. Darkening of the plug was observed at about 12 hpa although the plug was still easily dislodged. A dark-black plug was observed about 24 hpa and this plug was difficult but possible to dislodge by vigorous swirling of the water surrounding the animal. At 48 hpa the black plug was impossible to dislodge by swirling. The timing of these events was identical in all four *G. minus* populations as well as in *G. cohabitus*.

### Cave shrimp *Troglocaris anophthalmus* lost the ability to synthesize melanin at the wound site

**Figure 2:**
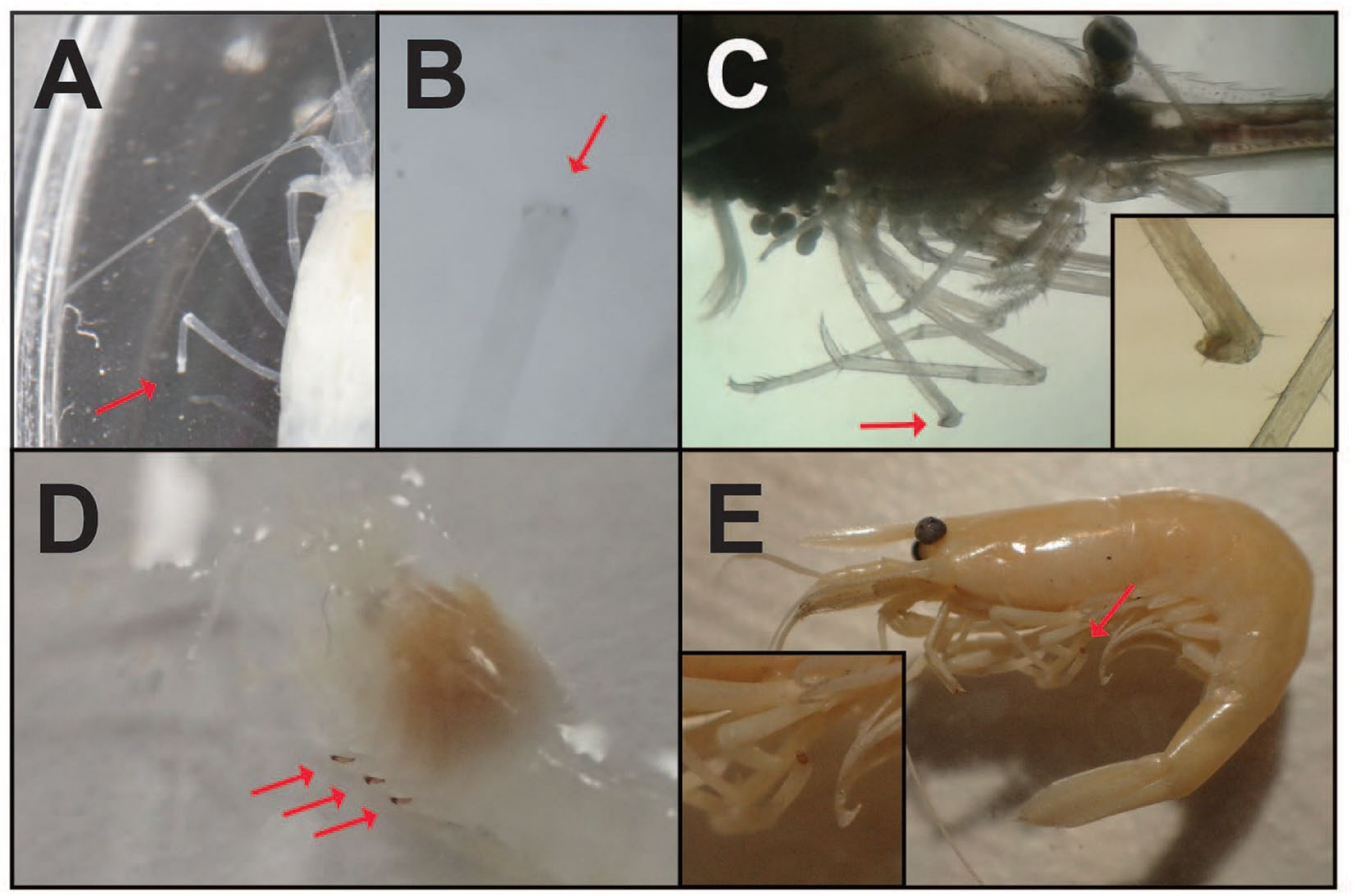
Albino cave shrimp *Troglocaris anophthalmus* lost the ability to synthesize melanin at the wound site (**A** and **B**, indicated by red arrow). Surface shrimp show melanized clot at the wound site (**C**). The albinoTexas cave shrimp *Palaemon antrorum* melanizes wound sites (**D**). An *Atyaephyra desmarestii* specimen (surface shrimp) shows a melanized wound (**E**). Insets in C and E are magnified images of injured limb. Photographs by the authors.

The albino atyid shrimp *Troglocaris anophthalmus* formed white plugs at wound sites but the plugs did not melanize even after four days (Fig. 2A and B). This was observed in all *Troglocaris* we collected from various geographic regions of Croatia (see Tables 1 and 2), and they belonged to phylogenetically distinct clades^27^. We further confirmed this observation by examining *Troglocaris* specimens in the collections of the Croatian Biospeleological Society in Zagreb (as of 6 July 2017). Research collections of albino cave-adapted crustaceans such as amphipods often contain specimens with dark spots at ends of broken appendages, presumably formed as part of the wound melanization reaction. We inspected 54 specimens from 17 caves spanning from northern Croatia to southern Herzegovina and found no dark coloration in any of the specimens with broken appendages. Therefore, we have high confidence that *T. anophthalmus* does not melanize its wounds. This loss of wound melanization in *Troglocaris* appears to be an apomorphy, because a closely related surface species, *Atyaephyra desmarestii*, also collected in Croatia, showed wound melanization (Fig. 2E). In addition, a surface marine shrimp in the genus *Crangon* in the family Crangonidae collected in Massachusetts, as well as both the surface-dwelling freshwater shrimp *Palaemon paludosus* and the cave-adapted albino shrimp *P. antrorum* from Texas also showed wound melanization (Fig. 2C and D). To our knowledge, this is the first documentation and, so far, the only example of loss of wound melanization as a derived condition associated with colonization of the subterranean environment.

### Wound melanization is present in all major arthropod lineages and is the ancestral state in Arthropods but has been convergently lost at least three times

**Figure 3:**
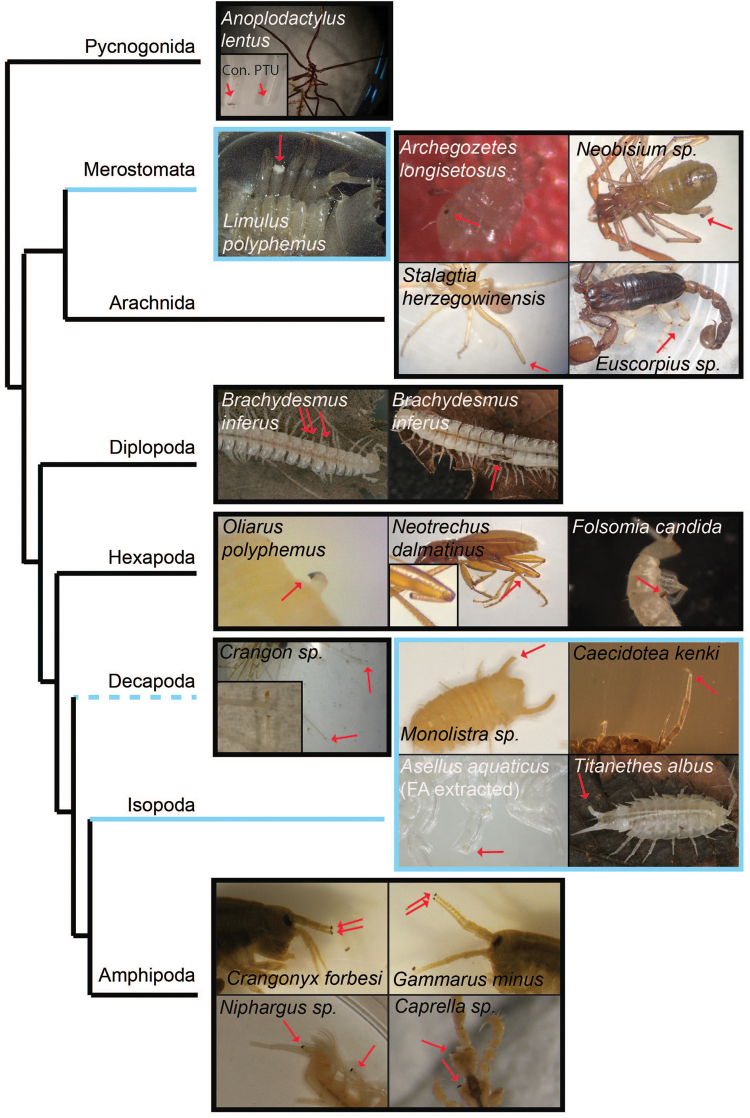
Wound melanization is present in most major arthropod lineages. Light blue lines indicate a loss of wound melanization in that lineage; dotted light blue indicates both phenotypes are present in that lineage. Arthropod relationships based on Legg *et al*.^62^. Photographs by the authors.

Wound melanization was present in the major arthropod lineages we tested, including Pycnogonida, Chelicerata, Myriapoda, and Pancrustacea, but it was absent in Merostomata and Isopoda (Table 1 and Fig. 3). The presence of the melanization response in the Pycnogonida, and in most of the lineages tested, suggests that wound melanization is an ancestral state in arthropods. This points to three independent losses of wound melanization among the groups we tested: in the atyid shrimp *T. anophthalmus*, in the merostome *Limulus polyphemus*, and in isopods. The situation with isopods is different from that of *T. anophthalmus* discussed above. All 15 species and clades, belonging to nine genera in four families, from both subterranean and especially surface environments, whether albino or not, lacked the response (Table 1). Thus, the loss of wound melanization appears plesiomorphic in isopods, and is likely to have occurred prior to colonization of the subterranean habitats and therefore not part of adaptation to the aphotic environment. Because no other merostome was investigated, no inferences can be made about whether lack of wound melanization was apomorphic for *L. polyphemus* or was lost earlier in the lineage.

## Discussion

The phenoloxidase reaction is one of the most effective immune mechanisms of invertebrates and it is present in many groups in addition to arthropods, including mollusks, annelids, echinoderms, tunicates and cephalocordates^14^. Melanin related enzymes and machinery can play a role in immunity in vertebrates as well. In zebrafish^28^ and *Astyanax mexicanus* (Bilandžija *et al*., in preparation) melanocytes migrate to the wound site in certain cases. There is evidence that melanin, melanosomes and melanocytes function as part of the innate immune system in humans as well^29,30^. Therefore, the role of melanin in immunity appears ancient and is present in different forms and to different extents throughout the animal kingdom. In arthropods melanin synthesis is a central mechanism of innate immunity and a major response to a variety of immune challenges^14,16,18,31^.

Most cave-adapted arthropods have lost body and eye pigmentation in the light depleted cave environment, and we wondered whether they have also lost the ability to synthesize melanin pigment as a part of the immune response. If coloration and other important functions of pigment are not essential for the survival of cave-adapted species, then we would expect the complete inactivation of pigment production pathways in cave-adapted arthropods. Alternatively, selection could either maintain a pigment synthesis pathway only in specific tissues or cell types, or maintain the parts of the pathway involved in defense processes but not in body pigmentation. Only a few studies aimed at understanding the mechanism of albinism in cave organisms at the molecular level have been conducted, and none have examined the relationship between loss of body pigmentation and wound melanization. In the Mexican cavefish *Astyanax mexicanus*, albinism was a result of loss of function deletions in the *oca2* gene that functions in the first step of melanin synthesis^4^. Similarly, in the cixiid planthoppers *Oliarus polyphemus* from Hawaii and *Trirhacus helenae* from Croatia, the loss of melanin was also due to disruption of the first step of melanin synthesis^2^.

We tested for cuticular wound melanization in multiple arthropod species spanning all known subphyla that have albino cave-adapted representatives. In addition to the cuticle, melanization occurs during wound healing of internal tissues in *Drosophila*, such as barrier epithelia of the epidermis, the trachael system and the gastrointestinal tract, and involvement of a host of different molecules, such as serpins and especially antimicrobial proteins have been demonstrated^31–35^. Therefore, results from this study are applicable only to wound melanization in the cuticle. Our data demonstrate for the first time that most albino cave-adapted arthropod species retain the ability to synthesize melanin during cuticular wound healing. This suggests that even if there is selection for albinism in cave animals (to conserve energy or to use precursors for alternative pathways), it does not affect the enzymatic machinery necessary for melanin synthesis needed in the immune response. Therefore, the melanin synthesis pathway is modular: some aspects of the pathway can be modified or inactivated while others remain intact. In the case of the cixiid planthopper, melanin synthesis pathway in the epidermis and cuticle is inactive^2^ and is activated after an immune challenge. On the contrary, in isopods melanin is synthesized for pigmentation of individual body parts but is not in response to wounding (see below). Decoupling of melanin synthesis between immune and body pigmentation contexts may be achieved by using different enzymes or different branches of the melanin synthesis pathway in different contexts. Alternatively, the same enzymes can be involved but specific functions are enabled by differential regulation, different activation pathways, or by their spatial localization.

The melanin synthesis pathway in arthropods is well categorized in insects^6^, and starts with hydroxylation of tyrosine into DOPA, catalyzed by tyrosine hydroxylase (TH) or phenoloxidase (PO) or both. The next step is a branch point where DOPA may be carboxylated to dopamine by dopadecarboxylase (DDC) or oxidized to dopaquinone by PO. Further downstream dopamine and dopaquinone are oxidized into dopamine melanin and dopamelanin, respectively^17,36^. There is evidence for using both different and the same enzymatic pathways for different functions of melanin. Many insect species possess multiple homologues of the PO genes (e.g. three PO genes in *Drosophila*, nine in *Anopheles gambiae*, 10 in *Aedes aegypti* ^37,38^). Subfunctionalization between different paralogues of PO has been demonstrated^7,39^. For example, in *Armigeres subalbatus* PO I is involved in melanization of microfilariae and PO II in hardening of egg chorion^7^. In *Tribolium castaneum* only *laccase2* is involved in cuticle tanning^40^. In *A. gambiae* laccases function in cuticle tanning and not in immunity^41^ but in *Anopheles sinensis laccase2* is involved in both cuticle tanning and immune response^42^. On the other hand, enzymes involved in cuticle darkening and sclerotization, such as phenylalanine hydroxylase, tyrosine hydroxylase, DOPA decarboxylase, and dopachrome conversion enzyme, separately or in various combinations, have been shown to be immune responsive^42–45^. Also, TH, DDC and *laccase2* are expressed in epidermal and cuticular tissues as well as in immune tissues such as fat body and hemocytes^42,45,46^. Therefore, melanization processes involved in cuticular wound healing, in barrier epithelial wound healing, and in body pigmentation may be independently controlled or encompass different enzymatic machinery.

The only cave-adapted animal we investigated that has lost the ability to melanize cuticular wounds is the cave shrimp *Troglocaris anophthalmus*. The loss of cuticular wound melanization is likely related to cave adaptation since all surface shrimp species we tested, including the close surface relative *Atyaephyra desmaresti* synthesizes melanin as a response to wounding. The cave-adapted albino shrimp, *Palaemon antrorum*, is also still able to melanize their wounds. Therefore, loss of cuticular wound melanization does not necessarily accompany cave adaptation in decapod shrimp, but is lineage specific for *T. anophthalmus*. *Troglocaris* shrimp formed non-melanized plugs at the wound sites and survived the amputations, suggesting they have developed alternative mechanisms for wound healing. Similarly, successful defense from some pathogens does not require PO activity in *Drosophila* and *Anopheles gambiae* ^18^, suggesting that melanization is not an indispensable defense strategy.

Reduction of metabolic rate is a common adaptation in many cave animals^1^ and specifically in cave-adapted compared to surface-dwelling amphipods^47^. The less cave-adapted *Gammarus troglophilus* showed a two- to four-fold higher oxygen consumption rate compared to the closely related and highly cave-adapted *G. acherondytes* ^47^. Our preliminary data using an intermittent flow respirometer system (Fong, unpublished data, in preparation) indicate a lower oxygen consumption rate in the two cave-adapted compared to the two surface-dwelling populations of *G. minus* used in this study, and they showed identical speeds of wound melanization. Although there is no data on oxygen consumption rate of *G. cohabitus*, it is sympatric with and is closely related to surface-dwelling *G. minus* ^26^, and may show a parallel reduction in oxygen consumption rate similar to the situation of *G. acherondytes* and *G. troglophilus*. Indeed, the timing of events during the melanization of wounds was nearly identical in most amphipod species used in this study regardless of habitat and higher taxonomic grouping, and they were unlikely to have identical metabolic rates, which strongly suggests that the speed of wound melanization is unaffected by differences in metabolic rates. One hypothesis for a potential evolutionary advantage of colonizing caves proposes reduced pressure on the immune system because of lower occurrence of parasites and pathogens (e.g.^23^, but see^48^). Our data does not support this hypothesis, because albino cave arthropods (except for *T. anophthalmus*) retain the wound melanization response at equal speeds as their closely related surface counterparts as we demonstrate in cave and surface *G. minus* populations.

To understand how important and widespread the melanization reaction is in wound healing we inflicted injuries on a range of arthropod species spanning all major subphyla. Our results showed that arthropods from diverse groups melanize wounds, suggesting that wound melanization was most likely already present in the last common ancestor of all Arthropoda. However, we found that wound melanization was lost in at least three lineages - the order Isopoda, the chelicerate *Limulus polyphemus*, and the atyid shrimp *T. anophthalmus*.

We tested 15 albino and non-albino isopod species and clades belonging to nine genera in four families from terrestrial and aquatic habitats in surface and subterranean environments, and none showed wound melanization. However, isopods have darkened mouthparts and tips of walking appendages, presumably due to melanin^49,50^ which contributes to the hardness of these structures. Moreover, we have observed melanized mouthparts and tips of dactyls of walking legs even in the cave-adapted albino isopods, meaning their melanin synthesis pathway is functional at least during intermolt stages. Furthermore, isopods can melanize and encapsulate parasites^51^ or invading bacteria^52^, indicating that melanin synthesis in isopods is independently regulated in response to different immune challenges. Similarly, different regulatory modules direct wound melanization and pathogen encapsulation in *Aedes aegypti* ^39^. Also, different signals induce PPO activation in different species. For instance, inner membrane lipids and not bacterial elicitors lipopolysaccharide nor peptidoglycan induce melanization in *Drosophila melanogaster*, whereas the opposite is true in the moth *Galleria mellonella* ^53^. In the mosquito *Armigeres subalbatus, Escherichia coli* were predominantly phagocytosed and *Micrococcus luteus* were mostly melanized^54^. Therefore, although the cause is unknown, only the cuticular wound melanization reaction is lost in isopods, indicating melanin synthesis is context dependent, being active in certain body parts and in response to some immune stimuli, but is inactive in the immune response during cuticular wound healing.

*Limulus polyphemus* also did not melanize wounds, even a week after limb amputation. We also checked the entire collection of horseshoe crabs in the Woods Hole Marine Biology Lab aquarium facility and found more than 10 individuals that had injuries and none of them had any dark pigmentation in the scar. We therefore concluded that *L. polyphemus* does not use melanin in wound healing.

A common trait of both *L. polyphemus* and isopods is that they lack the PO gene and that hemocyanins perform an analogous function in the immune response^55,56^. However, isopods can melanize encapsulated parasites, indicating that their melanin synthesis is functional. It is known that hemocyanins can be functionally transformed into phenoloxidase and can perform melanin synthesis^57,58^, that hemocyanins are also present in the cuticle and involved in sclerotization^59^, and that transformation of hemocyanins into immune functioning enzymes can be achieved similarly like PPO activation^56^. It is possible that the lack of wound melanization in *Limulus* and in isopods is due to different modes of activation of hemocyanins compared to phenoloxidases.

All chelicerate groups we tested (scorpions, spiders and pseudoscorpions) melanized wounds except for *L. polyphemus*. There are differing reports as to whether Chelicerata, or various chelicerate lineages, have PO genes^55,60,61^. The fact that arachnids melanize wounds suggests that either phenoloxidases are present in Chelicerates, or their hemocyanins are capable of synthesizing melanin as part of the wound healing process.

## Conclusions

We conclude that melanization is an important part of the cuticular wound healing process in arthropods. It is present in all major arthropod subphyla and was likely already present in the last common ancestor of all arthropods. Melanization of cuticular wounds is maintained even in aphotic subterranean environments where the loss of body and eye pigmentation is a convergent feature in diverse animal groups. Additionally, melanization reactions occur at similar rates in both cave and surface *G. minus*, despite cave populations having lower metabolic rates. To our knowledge, this is the first documentation of cuticular wound melanization in albino arthropods.

However, cuticular wound melanization has been lost in several arthropod lineages, including isopod crustaceans, the chelicerate *Limulus polyphemus*, and the cave shrimp *Troglocaris anophthalmus*. Therefore, melanization is not an indispensable process in cuticular wound healing. The loss of cuticular wound melanization in *T. anophthalmus* likely resulted from colonization of the subterranean environment because all other shrimp species we tested, including the most closely related surface relative, melanize wounds. This is the first documentation of loss of cuticular wound melanization as a derived condition associated with subterranean environments.

## References

[1] Culver, D. C. & Pipan, T. *The biology of caves and other subterranean habitats*. (Oxford University Press, 2009).

[2] Bilandžija, H., Ćetković, H. & Jeffery, W. R. Evolution of albinism in cave planthoppers by a convergent defect in the first step of melanin biosynthesis. *Evol. Dev*. **14** (2012). 10.1111/j.1525-142X.2012.00535.x PMC6169799 23017027

[3] Protas ME, Trontelj P, Patel NH (2011). Genetic basis of eye and pigment loss in the cave crustacean. Asellus aquaticus. Proc. Natl. Acad. Sci. USA.

[4] Protas ME (2006). Genetic analysis of cavefish reveals molecular convergence in the evolution of albinism. Nat. Genet..

[5] Ducrest A-L, Keller L, Roulin A (2008). Pleiotropy in the melanocortin system, coloration and behavioural syndromes. Trends Ecol. Evol..

[6] True JR (2003). Insect melanism: the molecules matter. Trends Ecol. Evol..

[7] Christensen BM, Li J, Chen C-CC, Nappi AJ (2005). Melanization immune responses in mosquito vectors. Trends Parasitol..

[8] Sugumaran M, Barek H (2016). Critical analysis of the melanogenic pathway in insects and higher animals. Int. J. Mol. Sci..

[9] Bilandžija H, Ma L, Parkhurst A, Jeffery WR (2013). A potential benefit of albinism in *Astyanax* cavefish: Downregulation of the oca2 gene increases tyrosine and catecholamine levels as an alternative to melanin synthesis. PLoS ONE.

[10] Kowalko JE (2013). Convergence in feeding posture occurs through different genetic loci in independently evolved cave populations of *Astyanax mexicanus*. Proc. Natl. Acad. Sci. USA.

[11] Gallo ND, Jeffery WR (2012). Evolution of space dependent growth in the teleost *Astyanax mexicanus*. PLoS ONE.

[12] Duboué ER, Borowsky RL, Keene AC (2012). β-adrenergic signaling regulates evolutionarily derived sleep loss in the Mexican cavefish. Brain. Behav. Evol..

[13] Milutinović B, Kurtz J (2016). Immune memory in invertebrates. Semin. Immunol..

[14] Cerenius L, Söderhäll K (2004). The prophenoloxidase-activating system in invertebrates. Immunol. Rev..

[15] Söderhäll K, Cerenius L (1998). Role of the prophenoloxidase-activating system in invertebrate immunity. Curr. Opin. Immunol..

[16] Ashida M, Brey PT (1995). Role of the integument in insect defense: pro-phenol oxidase cascade in the cuticular matrix. Proc. Natl. Acad. Sci. USA.

[17] Sugumaran M (2002). Comparative biochemistry of eumelanogenesis and the protective roles of phenoloxidase and melanin in insects. Pigment Cell Res..

[18] Cerenius L, Lee BL, Söderhäll K (2008). The proPO-system: pros and cons for its role in invertebrate immunity. Trends Immunol..

[19] Burmester T (2002). Origin and evolution of arthropod hemocyanins and related proteins. J. Comp. Physiol. B Biochem. Syst. Environ. Physiol..

[20] González-Santoyo I, Córdoba-Aguilar A (2012). Phenoloxidase: A key component of the insect immune system. Entomol. Exp. Appl..

[21] Chapman R. F. *The insects: structure and function*. (Cambridge University Press, 1998).

[22] Lee KP, Simpson SJ, Wilson K (2008). Dietary protein-quality influences melanization and immune function in an insect. Funct. Ecol..

[23] Tobler M, Schlupp I, García De León FJ, Glaubrecht M, Plath M (2007). Extreme habitats as refuge from parasite infections? Evidence from an extremophile fish. Acta Oecol..

[24] Ryazanova AD, Alekseev AA, Slepneva IA (2012). The phenylthiourea is a competitive inhibitor of the enzymatic oxidation of DOPA by phenoloxidase. J. Enzyme Inhib. Med. Chem..

[25] Carlini DB, Manning J, Sullivan PG, Fong DW (2009). Molecular genetic variation and population structure in morphologically differentiated cave and surface populations of the freshwater amphipod *Gammarus minus*. Mol. Ecol..

[26] Holsinger JR, Shafer J, Fong DW, Culver DC (2003). *Gammarus cohabitus*, a new species of subterranean amphipod crustacean (Gammaridae) from groundwater habitats in central Pennsylvania, USA. Subterr. Biol..

[27] Jugovic J, Jalžić B, Prevorčnik S, Sket B (2012). Cave shrimps *Troglocaris* s. str. (Dormitzer, 1853), taxonomic revision and description of new taxa after phylogenetic and morphometric studies. Zootaxa.

[28] Lévesque M, Feng Y, Jones RA, Martin P (2013). Inflammation drives wound hyperpigmentation in zebrafish by recruiting pigment cells to sites of tissue damage. Dis. Model. Mech..

[29] Mackintosh JA (2001). The antimicrobial properties of melanocytes, melanosomes and melanin and the evolution of black skin. J. Theor. Biol..

[30] Nappi AJ, Christensen BM (2005). Melanogenesis and associated cytotoxic reactions: Applications to insect innate immunity. Insect Biochem. Mol. Biol..

[31] Tang H, Kambris Z, Lemaitre B, Hashimoto C (2008). A serpin that regulates immune melanization in the respiratory system of *Drosophila*. Dev. Cell.

[32] Scherfer C (2008). Drosophila Serpin-28D regulates hemolymph phenoloxidase activity and adult pigmentation. Dev. Biol..

[33] Davis MM, Engström Y (2012). Immune response in the barrier epithelia: Lessons from the fruit fly D*rosophila melanogaster*. J Innate Immun.

[34] Lee, W.-J. & Miura, M. Mechanisms of systemic wound response in *Drosophila*. in *Current Topics in Developmental Biology* (ed. Galliot, B.) **108**, 153–183 (Academic Press, 2014). 10.1016/B978-0-12-391498-9.00001-2 24512709

[35] Nakhleh, J., El Moussawi, L. & Osta, M. A. The melanization response in insect immunity. in *Advances in Insect Physiology* (ed. Ligoxygakis, P.) **52**, 83–109 (Academic Press, 2017).

[36] Arakane, Y., Noh, M. Y., Asano, T. & Kramer, K. J. Tyrosine metabolism for insect cuticle pigmentation and sclerotization. in *Extracellular* Composite Matrices *in Arthropods* (eds Cohen, E. & Moussian, B.) 165–220 (Springer International Publishing, 2016).

[37] Zdobnov EM (2002). Comparative genome and proteome analysis of *Anopheles gambiae* and *Drosophila melanogaster*. Science.

[38] Chase, M. R. & Sugumaran, M. Genomic and cDNA sequence of prophenoloxidases from *Drosophila melanogaster*. in *Phylogenetic Perspectives on the Vertebrate Immune System. Advances in Experimental Medicine and Biology* (eds Beck, G., Sugumaran, M. & Cooper, E. L.) 349–362 (Springer, 2001). 10.1007/978-1-4615-1291-2_34 11419002

[39] Zou Z, Shin SW, Alvarez KS, Kokoza V, Raikhel AS (2010). Distinct melanization pathways in the mosquito *Aedes aegypti*. Immunity.

[40] Arakane Y, Muthukrishnan S, Beeman RW, Kanost MR, Kramer KJ (2005). Laccase 2 is the phenoloxidase gene required for beetle cuticle tanning. Proc. Natl. Acad. Sci. USA.

[41] Dittmer NT (2004). Characterization of cDNAs encoding putative laccase-like multicopper oxidases and developmental expression in the tobacco hornworm, *Manduca sexta*, and the malaria mosquito, *Anopheles gambiae*. Insect Biochem. Mol. Biol..

[42] Du M-H (2017). Suppression of *Laccase 2* severely impairs cuticle tanning and pathogen resistance during the pupal metamorphosis of *Anopheles sinensis* (Diptera: Culicidae). Parasit. Vectors.

[43] De Gregorio E, Spellman PT, Rubin GM, Lemaitre B (2001). Genome-wide analysis of the *Drosophila* immune response by using oligonucleotide microarrays. Proc. Natl. Acad. Sci. USA.

[44] Bartholomay LC (2004). Description of the transcriptomes of immune response-activated hemocytes from the mosquito vectors *Aedes aegypti* and *Armigeres subalbatus*. Infect. Immun..

[45] Qiao L (2016). Tyrosine hydroxylase is crucial for maintaining pupal tanning and immunity in *Anopheles sinensis*. Sci. Rep..

[46] Sideri M, Tsakas S, Markoutsa E, Lampropoulou M, Marmaras VJ (2008). Innate immunity in insects: surface-associated dopa decarboxylase-dependent pathways regulate phagocytosis, nodulation and melanization in medfly haemocytes. Immunology.

[47] WIilhelm FM, Taylor SJ, Adams GL (2006). Comparison of routine metabolic rates of the stygobite, *Gammarus acherondytes* (Amphipoda: Gammaridae) and the stygophile, *Gammarus troglophilus*. Freshw. Biol..

[48] Day J, Starkey DE, Gerken JE (2014). Prevalence of parasitism in the Grotto Sculpin (*Cottus* specus), a new species of cave-adapted fish from southeastern Missouri, USA. Subterr. Biol..

[49] Brehme KS (1941). The effect of adult body color mutations upon the larva of *Drosophila melanogaster*. Proc. Natl. Acad. Sci. USA.

[50] Gorman MJ, Arakane Y (2010). Tyrosine hydroxylase is required for cuticle sclerotization and pigmentation in *Tribolium castaneum*. Insect Biochem. Mol. Biol..

[51] Koehler AV, Poulin R (2010). Host partitioning by parasites in an intertidal crustacean community. J. Parasitol..

[52] Chevalier F (2011). The immune cellular effectors of terrestrial isopod *Armadillidium vulgare*: Meeting with their invaders, *Wolbachia*. PLoS ONE.

[53] Bidla G, Hauling T, Dushay MS, Theopold U (2009). Activation of insect phenoloxidase after injury: Endogenous versus foreign elicitors. J. Innate Immun..

[54] Hillyer JF, Schmidt SL, Christensen BM (2003). Hemocyte-mediated phagocytosis and melanization in the mosquito *Armigeres subalbatus* following immune challenge by bacteria. Cell Tissue Res..

[55] Jaenicke E (2009). Is activated hemocyanin instead of phenoloxidase involved in immune response in woodlice?. Dev. Comp. Immunol..

[56] Coates CJ, Nairn J (2014). Diverse immune functions of hemocyanins. Dev. Comp. Immunol..

[57] Decker H, Tuczek F (2000). Tyrosinase/catecholoxidase activity of hemocyanins: structural basis and molecular mechanism. Trends Biochem. Sci..

[58] Adachi K (2005). An oxygen transporter hemocyanin can act on the late pathway of melanin synthesis. Pigment Cell Res..

[59] Kuballa AV, Elizur A (2008). Differential expression profiling of components associated with exoskeletal hardening in crustaceans. BMC Genomics.

[60] Palmer WJ, Jiggins FM (2015). Comparative genomics reveals the origins and diversity of arthropod immune systems. Mol. Biol. Evol..

[61] Bechsgaard J (2016). Comparative genomic study of arachnid immune systems indicates loss of beta-1,3-glucanase-related proteins and the immune deficiency pathway. J. Evol. Biol..

[62] Legg DA, Sutton MD, Edgecombe GD (2013). Arthropod fossil data increase congruence of morphological and molecular phylogenies. Nat. Commun..

